# Individual-level and community-level determinants of cervical cancer screening among Kenyan women: a multilevel analysis of a Nationwide survey

**DOI:** 10.1186/s12905-017-0469-9

**Published:** 2017-11-15

**Authors:** Fentanesh Nibret Tiruneh, Kun-Yang Chuang, Peter Austin Morton Ntenda, Ying-Chih Chuang

**Affiliations:** School of Public Health, Taipei Medical University, 250 Wu-Hsing Street, Taipei City, Taiwan 110

**Keywords:** Cervical cancer, Pap test, Kenya, Sub-Saharan Africa, Women’s autonomy

## Abstract

**Background:**

Studies on the determinants of cervical cancer screening in sub-Saharan Africa have focused mostly on individual-level characteristics of cervical cancer screening. Therefore, in this study, we included both individual- and community-level indicators to examine the determinants of cervical cancer screening among Kenyan women.

**Methods:**

We analyzed data from the 2014 Kenya Demographic and Health Surveys. Our analysis focused on 9016 married women of reproductive age (15–49 years). We conducted multilevel analyses using generalized linear mixed models with the log-binomial function to simultaneously analyze the association of individual- and community-level factors with cervical cancer screening.

**Results:**

About 72.1% of women (*n* = 6498) knew about cervical cancer. Of these women, only 19.4% had undergone cervical cancer screening [58.24% Papanicolaou (Pap) test and 41.76% visual inspection]. Our multivariate analysis results indicated that the prevalence of cervical cancer screening was higher among women aged 35-49 years than women aged 15-24 years. The prevalence was also higher among women residing in the Central, Nyanza, and Nairobi regions than women residing in the Coastal region. Cervical cancer screening was more prevalent among women who had media exposure, had higher household wealth index, were employed, were insured, and had visit a health facility in 12 months than did their counterparts. The prevalence of Pap test history was 19% higher among women who had sexual autonomy than women who did not have sexual autonomy. The prevalence of Pap test history was also higher among communities comprised of higher proportions of women with sexual autonomy and higher education.

**Conclusions:**

Policies should emphasize increasing gender equality, improving education at the community level, providing employment opportunities for women, and increasing universal health insurance coverage. These focal points can ensure equity in access to health care services and further increase the prevalence of cervical cancer screening in Kenya.

## Background

Cervical cancer is the fourth most common cancer in women worldwide, with an estimated 528,000 new cases and approximately 275,000 deaths reported annually [1]. Moreover, 85% of the cases and most of the deaths occur in developing countries [2]. Cervical cancer incidence rates in sub-Saharan Africa (SSA) are the highest worldwide, and the disease is the most common cause of cancer death among women in this region [3, 4]. In Kenya, cervical cancer is the most prevalent cancer among women aged 15–44 years, with an estimated 4802 women diagnosed and 2451 deaths from the disease annually [5]. These elevated incidence and mortality rates can be attributed to the absence of the HPV vaccine and the low screening coverage [6].

Nearly all cervical cancer cases are caused by infection with high-risk types of human papillomavirus (HPV). Approximately 15 HPV types are associated with an increased risk of the disease; among the oncogenic HPV types, HPV16 and HPV18 are the most dangerous [7]. The implementation of an HPV vaccine program to prevent cervical cancer is one approach. However, to effectively see reduction rates in the incidence of cervical cancer, researchers and health professionals also recommend a cervical cancer screening program. Screening for cervical cancer is essential because vaccines do not treat existing HPV infections. Currently, a large proportion of women in low and middle income countries do not benefit from the HPV vaccine program. These women were either beyond the recommended age for the vaccines and/or already exposed to HPV [8].

Screening programs can save the lives of millions of women who develop precancerous lesions. For the early detection of cervical cancer and its precursor lesions, several screening modalities are now available, such as cytology or Papanicolaou (Pap) testing, visual inspection using acetic acid (VIA) or Lugol’s iodine (VILI), and HPV-test. The Pap test is a simple, safe, noninvasive, and effective method for detecting precancerous, cancerous, and noncancerous changes in the cervix and vagina [9]. Although the Pap test is effective, sustaining high-quality cytology-based programs is difficult in low-income countries because of the complex process of collection, preparation, staining, reading, and reporting and the delay between screening and provision of test results [10]. Therefore, in low-resource areas, cost-effective strategies that are inexpensive and of reliable quality are vital for preventing and intervening cervical cancer [11]. Common alternative screening tests are VIA and VILI. Although it has a lower specificity, VIA is still advocated as a screening method alternative to the Pap test in poorly resourced locations [12]. The attractive features of VIA and VILI include their low cost, simple administration, independence from laboratory services, and provision of real-time screening results, particularly in rural areas, where people travel for hours to visit a doctor. A screening method requiring fewer visits can largely increase acceptance and participation rates [11]. Thus, in low-income areas, particularly rural areas, VIA as a visual screening test is a promising alternative to the Pap test for the early detection of cervical cancer [13]. Cervical cancer screening programs have been available in Kenya, as a part of the Ministry of Health’s National Cervical Cancer Prevention Strategic Plan from 2002 to 2006. This program was implemented with the objective of increasing the use of the Pap test, VIA, and VILI among women [14]. Approximately 86% of women in Kenya have never been screened [15].

HPV testing is also feasible in low-resource settings. It is cost-effective and is well suited to address some of the barriers to implementing adequate screening programs in low resource settings [16]. Even though studies suggested that sampling by a clinician (in the context of a HPV testing program) should be the recommended method, HPV testing through the self-sampling method may be an acceptable option to reach women who do not or are not able to participate in the regular screening program [17, 18]. Therefore considering the lack of human resources, poor infrastructure, cost, long hospital queues and lack of quality cytopathology as the major barriers to cervical cancer screening in most SSA countries, self-sampling for HPV DNA test is an appropriate modality that can largely increase the coverage of cervical cancer screening [19].

A few studies have investigated the possible reasons for the low participation rates of women in cervical cancer screening programs in SSA. Lack of knowledge and awareness of cervical cancer are cited as the most common barriers to cervical cancer screening programs in SSA [20]. Other barriers include lack of financial resources, long distance to health facility, and lengthy waiting times to get an appointment for a Pap test [21, 22]. By contrast, high education level and white-collar occupation are positively associated with cervical cancer screening [23, 24]. Education equips women to have better knowledge toward the disease and thus increases the acceptance of cancer screening [25]. In addition, health insurance coverage and access to information through education and media are strongly and positively associated with screening experiences [26–28]. Some other researchers have investigated the influence of women’s decision-making autonomy on cervical cancer screening [29]. In most developing countries, gender norms and values continue to influence access to and utilization of sexual and reproductive health services [28, 30]. Gender norms may affect women’s mobility and decision-making power to access health care services. Women’s empowerment within the context of their household and relations with their partner can play a powerful role in their utilization of reproductive health services [31].

At the community level, low autonomy of women can influence their cervical cancer screening through cultural beliefs and practices. Most cultures in SSA consider women leaving their homes to seek health care to be unacceptable [32], particularly in rural area, where women living in communities are expected to not visit health care facilities alone; these women are less likely to use reproductive health care services [33]. By contrast, if the community norms support women’s own decision-making in health care seeking, women are more likely to use various health care services [34].

Most previous studies in SSA on the determinants of cervical cancer screening did not focus on the community-level characteristics. This study fills this gap by including both community-level indicators and individual-level characteristics. The objective of our study is to examine the determinants of cervical cancer screening among Kenyan women. We hypothesized that women who have higher levels of socioeconomic status and autonomy at both the individual and community levels, have health insurance, and feel that distance to health facilities is not a major problem are more likely to use cervical cancer screening than were their counterparts.

## Methods

### Data

This study used data from the most recent Kenya Demographic and Health Surveys (KDHS), conducted in 2014. The KDHS is a nationally representative dataset collected by the National Statistical Bureau in Kenya. The 2014 KDHS was designed to produce representative estimates for most of the survey indicators at the national level. A two-stage sampling design was applied that involved randomly selecting villages (clusters) in the first stage followed by randomly selecting households in the second stage. Questionnaires were pretested to ensure that the questions were clear and could be understood by respondents. Our analysis focused on 9016 married women of reproductive age (15–49 years) in 1588 clusters who were interviewed face to face about cervical cancer.

### Measures

#### Outcome variables

This study had two outcome variables: (1) cervical cancer screening in general and (2) the Pap test. Cervical cancer screening was measured in terms of whether respondents underwent any cervical cancer examination ever; respondents were specifically asked “Have you ever been tested or examined for cervical cancer?”(No/Yes). Respondents who answered “Yes” were then asked “What type of exam did you test?” (Pap test/Visual inspection).

#### Individual-level variables

Individual-level variables included the women’s age (15–24, 25–34, and 35–49 years), religion (Roman Catholicism, Protestant/other Christianity, Islam, and others), region (Coast, North Eastern, Eastern, Central, Rift Valley, Western, Nyanza, and Nairobi), place of residence (urban/rural), education level (no education, primary, secondary and higher), employment (no/yes), number of living children (0,1–2, 3–4, and ≥5), amount of media exposure (exposed to 0, 1, 2, and 3 types of media), health insurance coverage (no/yes), and whether the respondent visited a health facility in the last 12 months (no/yes). The wealth index was a composite score measured by household assets such as televisions, bicycles, materials used for house construction, water access types, sanitation facilities, and other characteristics related to wealth. Factor scores of household assets were generated through a principal component analysis and were then standardized and categorized into five quintiles (poorest, poor, middleclass, rich, and richest).

We measured three aspects of women’s autonomy: decision-making power in the household, sexual autonomy, and attitudes toward wife-beating at both individual and community levels. Decision-making power in the household was measured using the answers to the following five questions: the questions as to who decides matters pertaining to (a) the woman’s health (personal decision-making authority), (b) large household purchases (economic decision-making authority), (c) visits to friends or family (mobility decision-making authority), (d) food to be cooked each day, and (e) what to do with money the husband earns. Women who made all the aforementioned decisions, either alone or jointly with her husband, were categorized as having high decision-making autonomy, whereas the other women were categorized as having low decision-making autonomy. Sexual autonomy was measured according to the respondents’ agreement with some reasons in which a woman is justified to refuse sex with her husband. Attitudes toward wife-beating or domestic violence was measured on the basis of the following five hypothetical scenarios: (1) she goes out without telling him, (2) she neglects the children, (3) she argues with him, (4) she refuses to have sexual intercourse with him, and (5) she does not cook food properly. If a respondent agreed that her husband had a right to beat her in any of these five hypothetical scenarios, she was classified as having a favorable attitude toward domestic violence against women. However, if she did not agree with all of these hypothetical scenarios, she was classified as having an opposing attitude toward domestic violence against women.

#### Community-level variables

We included six community-level variables in our study, obtained by aggregating individual responses for each item to the community (cluster) level. The six community-level variables were the proportions of women who had high decision-making autonomy, had sexual autonomy, had favorable attitudes toward wife-beating, received secondary and higher education, perceived the distance to a health facility as a major problem, and had a nonpoor wealth index. Each variable was categorized into low, middle, and high on the basis of tertiles.

### Statistical analyses

We conducted series bivariate and multilevel analyses using SAS 9.4. We used the chi-squared test to examine the association of individual- and community-level characteristics with cervical cancer screening. We further conducted multilevel analyses using generalized linear mixed models with the log-binomial function to simultaneously analyze the relationships of individual- and community-level factors with the outcomes. Results of the multivariate relationships were expressed as Prevalence Ratio (PR) with 95% confidence intervals (95% CIs) [35, 36]. We first constructed a null model. We then included only the individual-level variables and then only community-level variables. Finally, we simultaneously included both individual- and community-level variables in the final model. We present only the final models. We also calculated intraclass correlation coefficients (ICCs), which represent the proportion of variance at the group level divided by the sum of the variances at the individual and group levels, to determine how well we adjusted the dependency of outcomes within communities. We examined the model fit measured using the Akaike information criterion (AIC). A lower AIC value represents a better model fit. We examined multicollinearity problems in the regression models by estimating the variance inflation factor and tolerance. All tolerance values were >0.1 and all variance inflation factor values were <10. Therefore, no multicollinearity problems were observed in the regression models.

### Ethical considerations

All data were obtained from the 2014 KDHS. Informed consent was obtained from each respondent before the interviews [12]. We obtained approval to use the data from the DHS repository (http://dhsprogram.com/data/available-datasets.cfm).

## Results

Approximately 28% of married Kenyan women had no previous knowledge of cervical cancer (Table 1). Among the women who knew about cervical cancer (72%), only 19.40% reported being tested for cervical cancer ever; among the tested women, only 58.24% had undergone the Pap test. Most women in our sample resided in rural areas, had completed primary education, and were employed. Furthermore, approximately 39% of the women had high decision-making autonomy at home. Almost half of all respondents had favorable attitudes toward wife-beating. Nearly 71.16% of all women had sexual autonomy. Only 18.27% women had health insurance.

**Table 1:** Characteristics of married women in the Kenya Demographic and Health Survey (KDHS)

	Percent
Individual-level Characteristics (*n* = 9014)	
Heard of cervical cancer	
No	27.90
Yes	72.10
Any kind of cervical cancer screening (*N* = 6498)	
No	80.60
Yes	19.40
Type of cervical cancer screening (*N* = 1262)	
Visual inspection	41.76
Pap smear	58.24
Socio-economic characteristics	
Women’s Age	
15-24	20.28
25-34	43.53
35-49	36.19
Religion	
Roman Catholic	18.83
Protestant/other Christian	64.59
Muslim	14.34
Others	2.24
Region	
Coast	12.49
North Eastern	5.82
Eastern	16.96
Central	10.12
Rift Valley	28.53
Western	8.85
Nyanza	14.38
Nairobi	2.8
Place of residence	
Urban	36.51
Rural	63.49
Women’s education level	
No Education	16.56
Primary	52.65
Secondary and higher	30.78
Women’s employment	
No	35.24
Yes	64.76
Number of living children	
0	4.95
1-2	36.30
3-4	32.86
5+	25.89
Amount of media exposure	
0	19.18
1	33.66
2	28.44
3	18.81
Wealth Index	
Poorest	24.67
Poor	19.22
Middle class	18.67
Rich	18.03
Richest	18.41
Women’s autonomy	
Women’s decision-making power at home	
Low	60.94
High	39.06
Sexual autonomy	
No	26.63
yes	71.16
Attitudes toward wife beating	
Opposing	50.13
Favorable	49.87
Health care access	
Covered by health insurance	
No	81.73
Yes	18.27
Visiting health facility in the last 12	
No	24.57
Yes	75.43
Community-level Characteristics (*n* = 1588)	
% of women with high decision-making autonomy	
Low	36.37
Medium	38.71
High	24.92
% of women with high sexual autonomy	
Low	30.07
Medium	30.86
High	39.08
% of women with favorable attitudes toward wife beating	
Low	48.21
Medium	22.35
High	29.41
% women with secondary and higher education	
Low	42.00
Medium	20.34
High	37.66
%of women who perceived the distance to a health facility as a major problem	
Low	46.91
Medium	23.56
High	29.53
% of women with non-poor wealth index	
Low	35.78
Medium	24.23
High	39.99

Table 2 presents the results of the bivariate analysis of the individual- and community-level characteristics and cervical cancer screening. All individual- and community-level variables were significantly associated with any type of cervical cancer screening in the bivariate analysis, except for the numbers of living children, whether the woman visit a health facility in the last 12 months, and the proportion of women in communities with high decision-making autonomy. In addition, the outcome of having undergone the Pap test ever was significantly associated with most individual-level and all community-level variables. Among individual-level variables, women’s religion, employment status, decision-making power at home, attitudes toward wife-beating, and whether the woman visited a health facility in the last 12 months were not significantly associated with a history of the Pap test.

**Table 2:** Bivariate associations of individual and community-level characteristics with cervical cancer screening history

	Any kind of cervical cancer screening history (*N* = 6498) %		Pap smear history (*N* = 1262) %	
Individual-level characteristics	No	Yes	No	Yes
Socio-demographic characteristics				
Women’s Age				
15-24	87.34	12.66*	53.10	46.90*
25-34	80.78	19.24	43.28	56.72
35-49	77.20	22.80	36.90	63.10
Religion				
Roman Catholic	78.44	21.56*	38.24	61.76
Protestant/other Christian	79.79	20.21	42.81	57.19
Muslim	93.70	6.30	32.14	67.86
Others	86.05	13.95	50.00	50.00
Region				
Coast	88.03	11.97*	41.57	58.43*
North Eastern	84.00	16.00	66.44	33.56
Eastern	81.55	18.45	41.94	58.06
Central	66.25	33.75	48.71	51.29
Rift Valley	82.99	17.01	43.25	56.75
Western	84.41	15.59	50.57	49.43
Nyanza	81.74	18.26	36.14	63.86
Nairobi	68.86	31.14	14.08	85.92
Place of residence				
Urban	76.60	23.40*	35.53	64.47*
Rural	83.30	16.70	47.26	52.74
Women’s education level				
No Education	94.29	5.71*	54.55	45.45*
Primary	83.33	16.67	48.03	51.97
Secondary and higher	74.43	25.57	35.15	64.85
Women’s employment				
No	85.60	14.40*	45.95	54.05
Yes	78.65	21.35	40.47	59.53
Number of living children				
0	80.87	19.13	45.61	54.39*
1-2	79.01	20.99	41.81	58.19
3-4	78.59	21.41	37.45	62.55
5+	86.41	13.59	50.00	50.00
Amount of media exposure				
0	90.62	9.38*	58.73	41.27*
1	85.87	14.13	44.26	55.74
2	79.93	20.07	40.96	59.04
3	69.58	30.42	38.16	61.84
Wealth Index				
Poorest	91.86	8.14*	37.84	62.16*
Poor	86.08	13.92	51.40	48.60
Middle class	82.80	17.20	55.56	44.44
Rich	78.55	21.45	42.14	57.86
Richest	68.30	31.70	31.13	68.87
Women’s autonomy				
Women’s decision-making power at home				
Low	82.24	17.76*	42.38	57.62
High	78.11	21.89	40.69	59.31
Sexual autonomy				
No	82.87	17.13*	50.00	50.00*
Yes	79.80	20.20	39.30	60.70
Attitudes toward wife beating				
Opposing	78.91	21.09*	39.95	60.05
Favorable	82.67	17.33	44.09	55.91
Health care access				
Covered by health insurance				
No	84.98	15.02*	47.06	52.94*
Yes	66.05	33.95	33.73	66.27
Visit health facility in the last 12				
No	82.05	17.95	87.83	12.17
Yes	80.00	20.00	88.95	11.05
Community-level characteristics(*n* = 1588)				
High decision-making autonomy ^a^				
Low	83.27	16.73	82.28	17.72*
Medium	80.54	19.46	45.36	54.64
High	79.09	20.91	41.39	58.61
% of women with high sexual autonomy				
Low	83.38	16.62*	90.96	9.04*
Medium	82.30	17.70	40.43	59.57
High	80.24	19.76	39.92	60.08
% of women with favorable attitudes toward wife beating				
Low	85.07	14.93*	46.35	53.65*
Medium	81.13	18.87	48.44	51.65
High	77.25	22.25	80.37	19.63
% women with secondary and higher education				
Low	87.87	12.13*	87.04	12.96*
Medium	80.25	19.75	33.33	66.67
High	76.67	23.33	38.50	61.50
%of women who perceived the distance to a health facility as a major problem				
Low	80.26	19.74*	76.46	23.54*
Medium	86.27	13.73	45.65	54.35
High	86.23	13.77	43.55	56.45
% of women with non-poor wealth index				
Low	89.74	10.26*	92.07	7.93*
Medium	85.71	14.29	52.24	47.76
High	73.31	26.69	40.82	59.18

* *P* < 0.05

The results of the multilevel analyses are presented in Table 3. The results of the null model for any type of cervical cancer screening outcome, (result not shown) demonstrated a significant variance in cervical cancer screening behavior between communities. About 30 % of the total variance in the cervical cancer screening behavior was at the community level (ICC = 0.31, *p* < 0.001).

**Table 3:** Multilevel analyses of factors associated with any type of cervical cancer screening (pap smear or visual inspection) and pap smear test to detect cervical cancer, among married women in Kenya

	Any type of cervical cancer screening (*n* = 6498).	Pap smear test (*n* = 1262).
Individual Characteristics	Adjusted PR (95% CI)	Adjusted PR (95% CI)
*Socio-Demographic and Economic Factors*		
Age		
15-24	1.00	1.00
25-34	1.18(0.97-1.44)	1.18 (0.89-1.58)
35-49	1.29 (1.09-2.97)*	1.38(1.04-1.85)*
Religion		
Roman Catholic	1.00	1.00
Protestant/other Christian	0.93(0.79-1.10)	0.95 (0.77-1.16)
Muslim	0.42(0.28-1.05)	1.13 (0.62-2.03)
Others	1.20(0.64-2.24)	0.52 (0.21-1.30)
Region		
Coast	1.00	1.00
North Eastern	1.18(0.34-4.13)	3.07 (0.69-5.68)
Eastern	1.32(0.91-1.92)	1.14(0.77-1.67)
Central	1.84(1.47-2.31)*	1.02(0.69-1.51)
Rift Valley	1.17 (0.93-1.48)	1.07(0.75-1.54)
Western	1.29 (0.96-1.74)	1.05(0.69-1.60)
Nyanza	1.40(1.10-2.76)*	1.17(0.80-1.69)
Nairobi	1.65(1.26-2.17)*	2.23 (1.49-4.09)*
Place of residence		
Urban	1.00	1.00
Rural	0.86(0.69-1.07)	0.97 (0.78-1.20)
Women’s education level		
No Education	1.00	1.00
Primary	1.47(0.98-2.23)	1.29 (0.66-2.54)
Secondary and higher	1.43(0.92-2.21)	1.22 (0.59-2.51)
Women’s employment		
No	1.00	1.00
Yes	1.21(1.08-1.39)*	1.35 (1.13-1.61)*
Number of living children		
0	1.00	1.00
1-2	0.95 (0.77-1.16)	1.09(0.77-1.54)
3-4	1.03 (0.83-1.28)	1.16(0.84-1.65)
5+	0.80 (0.62-1.03)	0.92(0.60-1.39)
Amount of media exposure		
0	1.00	1.00
1	1.05(0.81-1.36)	1.26 (0.83-1.91)
2	1.14(0.87-1.49)	1.06 (0.73-1.62)
3	1.36(1.02-1.81)*	0.84(0.58-1.29)
Wealth Index		
Poorest	1.00	1.00
Poor	1.37(1.03-2.17)*	1.08 (0.78-1.48)
Middle class	1.52(1.12-2.05)*	1.13 (0.81-1.58)
Rich	1.66(1.22-2.24)*	1.52 (1.25-2.45)*
Richest	2.02(1.48-2.77)*	2.60(1.78-3.79)*
*Women’s autonomy*		
Women’s decision-making power at home		
Low	1.00	1.00
High	1.08(0.94-1.24)	1.07 (0.82-1.40)
Sexual autonomy		
No	1.00	
Yes	0.91(0.77-1.07)	1.19 (1.01-1.45)*
Justifying wife beating		
Opposing	1.00	1.00
Favorable	1.03(0.88-1.19)	1.04 (0.78-1.39)
*Health care access*		
Covered by health insurance		
No	1.00	1.00
Yes	1.62(1.45-1.83)*	2.05 (1.70-2.47)*
Visit health facility in the last 12		
No	1.00	1.00
Yes	1.20(1.03-1.40)*	0.90 (0.76-1.07)
Community- level characteristics		
High decision-making autonomy at home ^a^		
Low	1.00	1.00
Medium	1.01(0.79-1.29)	1.08 (0.79-1.47)
High	1.07(0.84-1.35)	0.90 (0.66-1.23)
% of women with high sexual autonomy		
Low	1.00	1.00
Medium	1.10(0.85-1.43)	0.98 (0.78-1.24)
High	0.92(0.70-1.121)	1.90 (1.35-2.67)*
% of women with favorable attitudes toward wife beating		
Low	1.00	1.00
Medium	1.04(0.82-1.32)	1.36 (0.97-1.90)
High	1.03(0.79-1.34)	0.99(0.71-1.40)
% of women with secondary and above education		
Low	1.00	1.00
Medium	0.88(0.68-1.15)	1.37 (0.99-1.89)
High	1.01(0.78-1.32)	*1.32(1.12-1.79)*
% of women who perceived the distance to a health facility as a major problem		
Low	1.00	1.00
Medium	1.04(0.86-1.42)	1.32 (0.90-1.92)
High	*1.03(0.99-1.90)*	1.14 (0.87-1.48)
% of women with non-poor wealth index		
Low	1.00	1.00
Medium	1.03(0.77-1.37)	0.86 (0.58-1.26)
High	1.06(0.77-1.47)	0.74 (0.55-1.01)
ICC	0.23*	0.10*
AIC	8852.95	5484.54

*PR* prevalence ratio, *CI* confidence interval, *ICC* intraclass correlation coefficient, *AIC* Akaike information criterion. * Significant at *P*-value <0.05,

^a^Percentage of women with high decision-making autonomy at home

Italic*P < 0.05

The analysis of only individual-level variables, revealed that women’s age, religion, region, education level, employment, amount of media exposure, household wealth index, visiting a health facility, and health insurance were significantly associated with use of any type of cervical cancer screening; the ICC indicated that 29% of the variation in cervical cancer screening was attributable to among community differences (ICC = 0.29, p < 0.001) (results not shown).

In the final model (Table 3), included both the individual- and community-level characteristics. The results showed that the prevalence of any type of cervical cancer screening was 1.29 times higher among older women than among younger women (APR = 1.29; 95% CI = 1.09–2.97). The prevalence was also higher among women residing in the Central, Nyanza, and Nairobi regions than among women residing in the Coastal region (APR = 1.84, 95% CI 1.47-2.31; APR = 1.40, 95% CI = 1.10–2.76; and APR = 1.65, 95% CI = 1.26–2/17, respectively). Regarding employment, any type of cervical cancer screening was 1.21 times more prevalent among employed women when compared to unemployed women (APR = 1.21; 95% CI = 1.08-1.39). Media exposure was also positively associated with any type of cervical cancer screening. The prevalence ratio for women who had exposure to three types of media was 1.36 compared to women who had no exposure to any media (APR = 1.36; 95% CI = 1.02-1.81). The prevalence ratio for women from the richest households was 2.02 compared with women from the poorest households (APR = 2.02; 95% CI = 1.48-2.77). Even after the inclusion of both individual- and community-level variables, the variation in cervical cancer screening behavior between communities remained significant; as shown by the estimated ICC, 23% of the variability in any type of cervical cancer screening was attributable to community differences (ICC = 0.23, *P* < 0.001).

The multilevel analyses of factors associated with the utilization of Pap test; the results for the null model indicated that 40% of the total variance in Pap test was accounted by between-community variations (ICC = 0.40, *P* < 0.001) (results not shown). The analysis of only individual-level variables revealed that the prevalence of Pap test was higher among the women who were older, resided in Central and Nairobi regions, were employed, had rich and the richest wealth index scores, had sexual autonomy, and had health insurance coverage than were their counterparts. The ICC indicated that 17% of the variation in the utilization of Pap test was attributable to community differences (ICC = 0.17, P < 0.001) (results not shown).

The final model was presented in Table 3. After included both individual and community-level variables in the final model, age, residence in the Nairobi region, employment, wealth index, sexual autonomy, and health insurance coverage were significantly associated with the prevalence of Pap test history (APR = 1.38, 95% CI = 1.04-1.85; APR = 2.23, 95% CI = 1.49-4.09; APR = 1.35, 95% CI = 1.13-1.61; APR = 2.60, 95% CI = 1.78-3.79; APR = 1.19, 95% CI = 1.01-1.45; and APR = 2.05, 95% CI = 1.70-2.47, respectively). The prevalence of Pap test history was higher among communities comprised of higher proportions of women with sexual autonomy and higher education (APR = 1.90, 95% CI = 1.35-2.67 and APR = 1.32, 95% CI = 1.12-1.79, respectively) than the counterparts. The estimated ICC indicated that 10% of the variability in Pap test history was attributable to community differences (ICC = 0.10, *P* < 0.001).

## Discussion

Our study contributes to the understanding of factors associated with cervical cancer screening in Kenya, where the prevalence of this screening remains low [24]. To our knowledge, this is the first study to assess both the individual- and community-level factors associated with cervical cancer screening in Africa. Our findings reveal that a significant number of women (80.6%) who had knowledge of cervical cancer did not use screening services; in addition, approximately 28% of women in Kenya had no prior knowledge of cervical cancer. Lack of knowledge, younger age, lack of income, fear of the Pap test, and lack of access to screening services were significantly associated with low cervical cancer screening rates [37, 38]. This suggests that cervical cancer screening programs can incorporate self-sampling HPV DNA tests. Recent studies have recommended that cervical cancer screening programs that incorporate self-sampling and HPV DNA tests are feasible, and may significantly improve uptake of cervical cancer screening in SSA [19]. Our study also showed that the prevalence of any type of cervical cancer screening was higher among those residing in the Central and Nyanza regions, while the prevalence of using a Pap test was higher in the Nairobi region. A possible explanation for the regional variation observed is that the Central, Nyanza, and Nairobi regions characteristically have higher socioeconomic status, less cultural conservatism, and easier access to health care services [39, 40].

Corroborating the results of similar studies, our results demonstrate that women’s employment and household wealth were positively associated with cervical cancer screening [27, 41, 42]. The lower prevalence of cervical cancer screening among unemployed and poorer women may indicate financial burden, which is a barrier to accessing cervical cancer screening services. Employed women were more likely to undergo cervical cancer screening because this group of women is most likely to own private health insurance [27].

Our study determined that both individual- and community-level women’s sexual autonomy had a positive influence on Pap testing behavior, indicating that gender inequality, as assessed through low sexual autonomy, can affect cervical cancer screening. Studies have indicated that women’s sexuality is much more controlled than men’s in most developing countries, where women are perceived as passive and powerless and societies describe sex as primarily a male domain [43, 44]. Furthermore, several myths and misconceptions related to women’s sexual and reproductive health after undergoing screening (e.g., cervical cancer screening reduces sexual satisfaction for men and women) have been widely accepted [45, 46]; consequently, this can cause various health problems. Our results indicate that empowering women to control their own lives and make their own decisions regarding their sexual and reproductive health is necessary [47].

Women who resided in communities comprising a higher proportion of women with secondary and higher education were likely to have a history of the Pap test. This finding is consistent with those of previous study results, suggesting that a community with a high concentration of educated women can increase the utilization of health care services including cervical cancer screening [48, 49]. Education is frequently associated with increased access to health care services and more knowledge regarding health behavior. Increasing the proportion of educated women may facilitate the dissemination of knowledge to those with lower education, aiding them in accessing health services through informal social networks and contacts.

Contradicting with our hypothesis and previous study findings [27, 50], we found a marginal positive association between the proportion of women that perceived distance to a health facility as a major problem and cervical cancer screening. The possible explanation for this unexpected result is that women who live in rural and/or remote areas have low expectations of health services, and thus judge the distance to a health facility as not a major problem. Other logistical barriers such as lack of transportation and lack of finance to access screening services are influential factors that can further influence screening behavior [21]. However, these variables are not available in our data set (KDHS). Future research should further include these potential logistical factors associated with cervical cancer screening in the study design and analysis.

As anticipated, health insurance coverage was strongly associated with both primary and secondary outcomes. Our finding regarding the association between health insurance and screening use is consistent with the results of previous studies [51, 52]. Our study results prove that the adoption of a universal health insurance scheme ensuring equity in access to health care can largely enhance the possibility of cervical cancer screening use [27]. Cost is one of the main barriers to obtaining a cervical cancer screening among women in resource-constrained countries [21, 50]. In a region where the poverty is high, emergency needs are given greater priority to out-of-pocket payments than preventive services [53]. Consequently, health insurance coverage may potentially reduce the financial burden for women to access preventive health care services, including cervical cancer screening.

Consistent with other studies, our study suggested that visiting a health facility in the last 12 months is positively associated with cervical cancer screening behavior. Having a usual source of care is important for women’s access to screening services [53, 54]. Particularly in resource-poor settings, contact with health professional workers while visiting a health facility can increase women’s exposure to related health knowledge and encourage women to undertake preventive services [55]. Prior studies also demonstrated that a health provider’s recommendation was consistently found to be a strong predictor of completion of cervical cancer screening [56].

Our study has some potential limitations. First, the cross-sectional study design limited our ability to draw causal inferences for the association of individual- and community-level factors with cervical cancer screening. Second, because of the limited number of variables collected by the KDHS, we could not examine a full array of factors related to cervical cancer screening, particularly cultural and supply-side factors including health service quality and other factors related accessibility to the services. Third, our community measures were based on aggregating individual responses to the community level, which may increase the likelihood of misclassifying individuals into inappropriate administratively defined boundaries (clusters).

## Conclusion

Our study determined that both individual- and community-level factors influence cervical cancer screening behavior. Specifically, geographical distribution of medical resources, exposure to health information through media, employment opportunities, health insurance coverage, and women’s own sexual autonomy, as well as women’s sexual autonomy and education at the community level, all contribute to their screening behavior. Our findings provide suggestions for future studies to address these factors associated with cervical cancer screening rates in Kenya.

### Implications for practice and/or policy

Our results suggest that the adoption of policies promoting access to information about the benefit of cervical cancer screening through media and improve gender equality can empower women to use screening services. Employment programs should aim to provide employment opportunities among women. Establishing income-generating programs for women may increase their intention toward and actual use of cervical cancer screening. Moreover, the influence of extending insurance overage could be substantial. Health insurance coverage can potentially reduce out-of-pocket health expenses for women and empower them financially to demand and be able to use health services. Health policymakers should also address the issue of geographical inequalities in screening behavior; this can be achieved through approaches such as increasing health facility and medical personnel in rural areas to minimize geographical inequality. Public health programs must target young adults to inform them about the benefits of early detection of cervical cancer and motivate them to initiate preventive behavioral changes. In addition, to improve cervical cancer screening coverage and achieve optimal protection, HPV testing by using self-collected samples is a plausible modality for cervical cancer screening in future policy development.

## Abbreviations

AIC: Akaike information criterion
APR: Adjusted prevalence ratio
CI: Confidence interval
DNA: Deoxyribon ncleic acid
HPV: Human papilloma virus
ICC: Intraclass correlation coefficient
KDHS: Kenya demographic health survey
Pap: Papanicolaou
SAS: Statistical analysis software
SSA: Sub-Saharan Africa
VIA: Visual inspection using acetic acid
VILI: Visual inspection using Lugol’s iodine
